# Development and characterization of the first dsRNA-resistant insect population from western corn rootworm, *Diabrotica virgifera virgifera* LeConte

**DOI:** 10.1371/journal.pone.0197059

**Published:** 2018-05-14

**Authors:** Chitvan Khajuria, Sergey Ivashuta, Elizabeth Wiggins, Lex Flagel, William Moar, Michael Pleau, Kaylee Miller, Yuanji Zhang, Parthasarathy Ramaseshadri, Changjian Jiang, Tracey Hodge, Peter Jensen, Mao Chen, Anilkumar Gowda, Brian McNulty, Cara Vazquez, Renata Bolognesi, Jeffrey Haas, Graham Head, Thomas Clark

**Affiliations:** Monsanto Co., 700 Chesterfield Parkway West, Chesterfield, Missouri, United States of America; University of Kentucky, UNITED STATES

## Abstract

The use of dsRNA to control insect pests via the RNA interference (RNAi) pathway is being explored by researchers globally. However, with every new class of insect control compounds, the evolution of insect resistance needs to be considered, and understanding resistance mechanisms is essential in designing durable technologies and effective resistance management strategies. To gain insight into insect resistance to dsRNA, a field screen with subsequent laboratory selection was used to establish a population of DvSnf7 dsRNA-resistant western corn rootworm, *Diabrotica virgifera virgifera*, a major maize insect pest. WCR resistant to ingested DvSnf7 dsRNA had impaired luminal uptake and resistance was not DvSnf7 dsRNA-specific, as indicated by cross resistance to all other dsRNAs tested. No resistance to the *Bacillus thuringiensis* Cry3Bb1 protein was observed. DvSnf7 dsRNA resistance was inherited recessively, located on a single locus, and autosomal. Together these findings will provide insights for dsRNA deployment for insect pest control.

## Introduction

Transgenic maize expressing *Bacillus thuringiensis* (Bt) proteins has been rapidly adopted on farms across the Midwestern U.S. Corn Belt to control WCR, *Diabrotica virgifera virgifera LeConte* and other corn rootworm (CRW) species[1, 2]. However, at least some resistance has been reported to all five Bt proteins currently used to control WCR[3–8], emphasizing the need for alternative management tools.

Since the first report of expressing insecticidal dsRNA in plants[9], the use of dsRNA to control insect pests via the RNA interference (RNAi) pathway has been explored by numerous researchers globally[10–15]. DvSnf7 dsRNA-expressing maize (*Zea mays* L.) targeting Western corn rootworm (WCR) was the first insecticidal dsRNA-expressing plant registered by US EPA (SmartStax Pro, 2017) and is scheduled to be commercialized by the end of the decade. The *D*. *virgifera* Snf7 (DvSnf7) protein is a class E vacuolar sorting protein belonging to the ESCRT (Endosomal Sorting Complex Required for Transport)-III complex, shown to be essential for transmembrane protein sorting[16]. Upon consumption, the plant-produced DvSnf7 dsRNA is recognized by the CRW’s RNA interference (RNAi) machinery resulting in down-regulation of the targeted DvSnf7 gene leading to WCR death[16, 17]. DvSnf7 dsRNA has been shown to control WCR at commercially-accepted dose and represents an insecticidal mode of action (MOA) different from that of Bt, and therefore can control Bt-resistant WCR[18].

With every new class of insect control compounds, the evolution of insect resistance needs to be considered, especially for WCR, because of its history of evolving resistance to numerous control strategies[4, 6, 19–21]. Understanding resistance mechanisms is essential in designing durable technologies and effective resistance management strategies, but there currently is no known case of insect resistance to dsRNA. To gain insight into dsRNA resistance in insects, we used a field screen with subsequent laboratory selection with maize expressing DvSnf7 dsRNA to establish an insect colony of dsRNA-resistant WCR.

## Results

### Establishing a WCR population resistant to maize expressing DvSnf7 dsRNA

**Fig 1:**
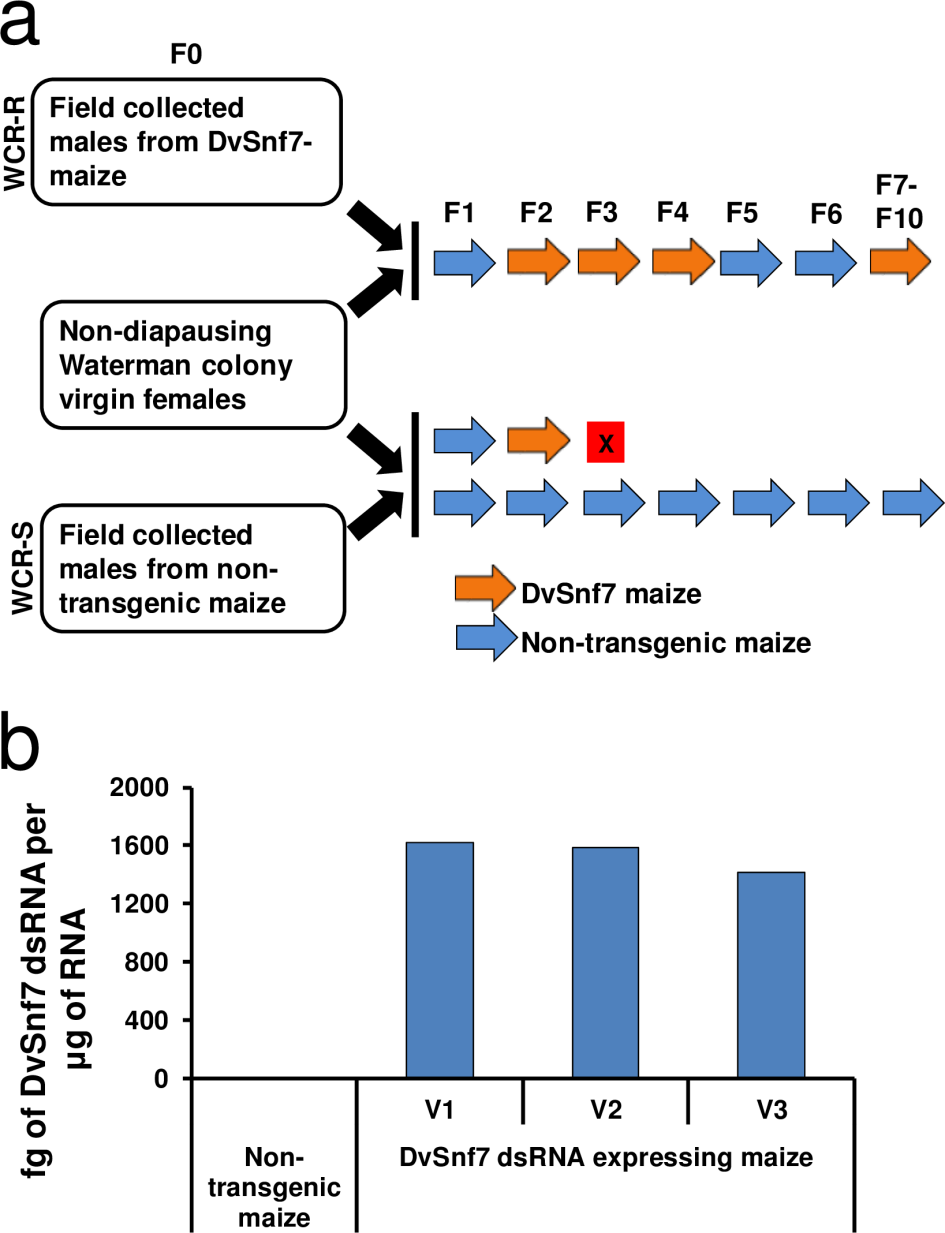
Establishing a WCR colony resistant to DvSnf7 dsRNA. (**a**) Selection regime used to establish DvSnf7 dsRNA resistant (WCR-R) and susceptible (WCR-S) colonies. A total of 350 beetles were collected from field-tented plots containing maize plants expressing DvSnf7 dsRNA for WCR-R, and 500 beetles from the surrounding area containing non-transgenic maize for WCR-S. Field collected males were allowed to mate with virgin females from the non-diapausing Waterman lab colony to produce non-diapausing colonies. From F1 onwards, beetles were sib-mated and reared on either DvSnf7 or non-transgenic maize roots. From F11, the WCR-R colony was selected on DvSnf7 maize every other generation. (**b**) Expression of DvSnf7 dsRNA in DvSnf7 maize roots (ZM_S295399) at three vegetative stages (V1, V2 and V3). Non-transgenic maize roots were used as a control. Bar graph, median. DvSnf7 dsRNA expression in the DvSnf7 dsRNA maize was not significantly different (*p* = 0.402) within developmental stages. Root samples were collected from maize grown in root mats used for rearing all generations of both colonies in growth chambers.

To establish a field-relevant WCR colony resistant to DvSnf7 dsRNA (WCR-R), a tented plot containing about 3,360 transgenic maize plants expressing only DvSnf7 dsRNA (DvSnf7 maize) was planted in a field known to have high WCR pressure near Waterman, IL. A comparable susceptible unselected WCR population (WCR-S) was established from non-transgenic (NT) maize planted in the area surrounding the tent. A total of 350 and 500 adult beetles were collected from DvSnf7 and NT maize, respectively. Both WCR-R and WCR-S populations were crossed in the laboratory with a non-diapausing WCR colony[22] to generate non-diapausing (ND) populations to increase the number of insect generations per year and thereby accelerate the resistance selection process. Selection regimes applied to both populations are described in Fig 1A. The expression of DvSnf7 dsRNA in DvSnf7 maize roots used for selection ranged from 1,593–2,192 fg / μg RNA, and was not significantly different (*p* = 0.402) within the maize developmental stages tested (Fig 1B).

**Fig 2:**
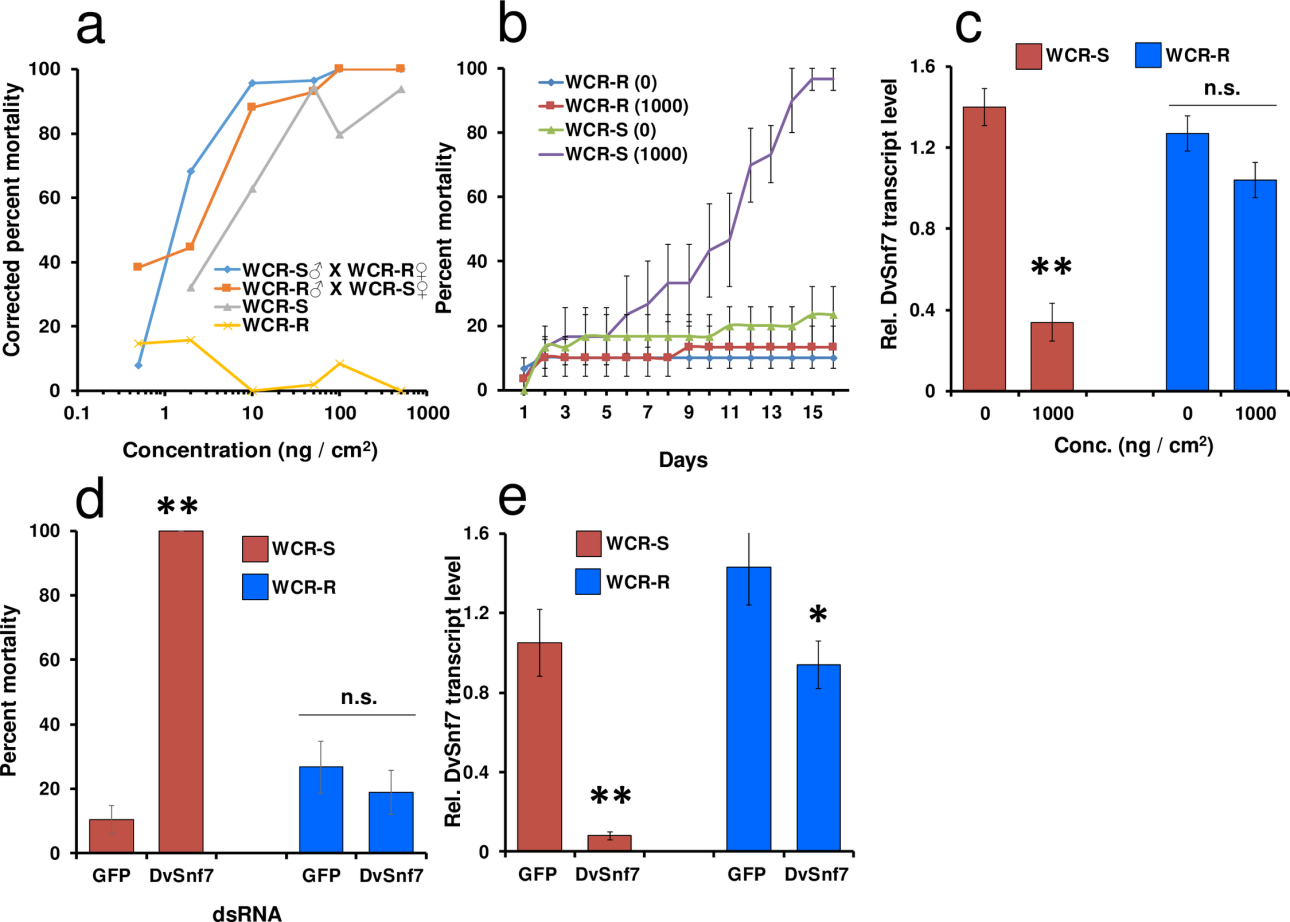
WCR-R larvae and beetles are resistant to DvSnf7 dsRNA and inheritance of DvSnf7 dsRNA resistance is recessive. (**a**) Concentration–response curves of WCR-S, WCR-R neonates and F1 neonates from WCR-R and WCR-S reciprocal crosses. Each cross was bulk-mated and consisted of 80 males and 80 females. Each population was assayed using 5–6 concentrations (*n* = 105–254). (**b**) Percent beetle mortality from WCR-R and WCR-S when exposed to artificial diet overlaid with DvSnf7 dsRNA (1000 ng /cm^2^) or water (0 ng /cm^2^). Assay was conducted one time with three replications, each consisting of 10 beetles (*n* for all treatments = 30). (**c**) DvSnf7 transcript levels determined using RT-qPCR in beetles fed for 72 hrs on artificial diet overlaid with DvSnf7 dsRNA or water (*n* = 7–8). (**d**) Percent beetle mortality from WCR-R and WCR-S when injected with 25 ng of either DvSnf7 or GFP dsRNA. Mortality was recorded 10 d after injection. Assay was conducted two times each with three replications (*n* = 30–48). (**e**) DvSnf7 transcript levels determined using RT-qPCR in beetles injected with 25 ng of DvSnf7 or GFP dsRNA. After injections beetles were allowed to feed on untreated artificial diet for 72 h and collected for transcript analysis (*n* = 3–8). (c to e) Means ± SEM. **p < 0.0001, *p = 0.042.

To determine the level and degree of dominance of DvSnf7 dsRNA resistance in WCR-R larvae, reciprocal crosses between WCR-R and WCR-S beetles were made in the laboratory and F_1_ neonates (along with WCR-R and WCR-S neonates) were exposed to increasing concentrations of purified DvSnf7 dsRNA to establish concentration response curves (Fig 2A). LC_50_ values for WCR-S, WCR-S males X WCR-R females, and WCR-S females X WCR-R males were 3.70, 1.42, and 1.48 ng /cm^2^, respectively (Table 1). The LC_50_ value for WCR-R could not be calculated because mortality never reached 50% even at the highest concentration tested (500 ng /cm^2^). Based on these LC_50_ values (assuming an LC_50_ value for WCR of at least 500 ng / cm^2^), there was a ≥ 130-fold difference in susceptibility to DvSnf7 dsRNA between WCR-R and WCR-S. Mortality curves generated in the bioassay indicate that resistance to DvSnf7 dsRNA was recessive and autosomal (Fig 2A). Dominance (D) was calculated using the formula of Stone[23], resulting in a value of -1.0, indicating resistance was completely recessive.

**Table 1:** Concentration response of WCR-R, WCR-S, and reciprocal crosses of each WCR population to DvSnf7 dsRNA overlaid on artificial diet.

Population	N^1^	Slope ± SE	LC_50_ (95% FL)^2^	Resistance ratio^3^	χ2^4^	*p* value^5^
WCR-S	105	1.39 ± 0.54	3.70 (0–15.8)	—	13.5 (11)	0.2616
WCR-R	253	NC^6^	>500	>130	—	—
WCR-S ♀ x WCR-R ♂	139	2.05 ± 0.61	1.48 (0.10–4.40)	0.40	9.2 (16)	0.9047
WCR-S ♂ x WCR-R ♀	254	3.26 ± 0.83	1.42 (0.56–2.66)	0.38	22.2 (28)	0.7734

^1^Total number of insects over all concentrations.

^2^DvSnf7 dsRNA (ng / cm2) after 12 days.

^3^Resistance ratio was calculated as LC_50_ value / LC_50_ value of WCR-S.

^4^Likelihood Ratio Chi-Square value and the degrees of freedom.

^5^*p* value: Goodness-of-Fit test *p* value based on Likelihood Ratio test.

^6^Not calculated as LC_50_ never reached 50%.

To determine if WCR larvae resistant to DvSnf7 dsRNA are still resistant as beetles, WCR-R and WCR-S beetles were fed artificial diet surface-treated with DvSnf7 dsRNA or water (Fig 2B). Almost 100% mortality was observed for WCR-S beetles fed diet treated with DvSnf7 dsRNA (1000 ng /cm^2^) compared to 23% mortality on untreated diet. Mortality for WCR-R beetles on treated and untreated diet remained below 15%. Beetles from all treatments were collected after 72 h of feeding and DvSnf7 transcript levels were evaluated using RT-qPCR. There was a significant reduction (4.1-fold) (*p* < 0.0001) in DvSnf7 transcript levels in WCR-S beetles fed treated diet as compared to untreated diet, but no significant reduction (*p* = 0.074) in DvSnf7 transcript levels were observed in WCR-R beetles exposed to treated or untreated diet (Fig 2C). These results demonstrate that not only WCR-R larvae are resistant to DvSnf7, but WCR-R beetles as well.

Beetles from both colonies were injected with 25 ng DvSnf7 or green fluorescent protein (GFP) dsRNA to determine if injecting DvSnf7 dsRNA in WCR-R would cause mortality and DvSnf7 transcript suppression. We observed significantly greater mortality (100%) (*p* < 0.0001) in WCR-S beetles injected with DvSnf7 dsRNA compared to WCR-S beetles injected with GFP dsRNA (10.4%), while mortality in WCR-R beetles injected with DvSnf7 (18.8%) or GFP (26.7%) dsRNA was not significantly different (*p* = 0.768) (Fig 2D). DvSnf7 transcript levels in beetles from both WCR-S and WCR-R were also measured 72 h after dsRNA injection (Fig 2E). We observed a significant reduction (13.1-fold; *p* < 0.0001) in DvSnf7 transcript levels in WCR-S beetles injected with DvSnf7 dsRNA compared to beetles injected with GFP dsRNA. In contrast, there was only a 1.5-fold significant reduction (*p* = 0.042) in DvSnf7 transcript levels in WCR-R beetles injected with DvSnf7 dsRNA compared to beetles treated with GFP dsRNA. However, a 1.5-fold reduction in DvSnf7 transcript level was not sufficient to cause mortality in WCR-R beetles, as we observed no difference in mortality in WCR-R beetles injected with DvSnf7 or GFP dsRNA (Fig 2D).

### WCR-R is cross resistant to other dsRNAs but not to a Bt Cry protein

**Fig 3:**
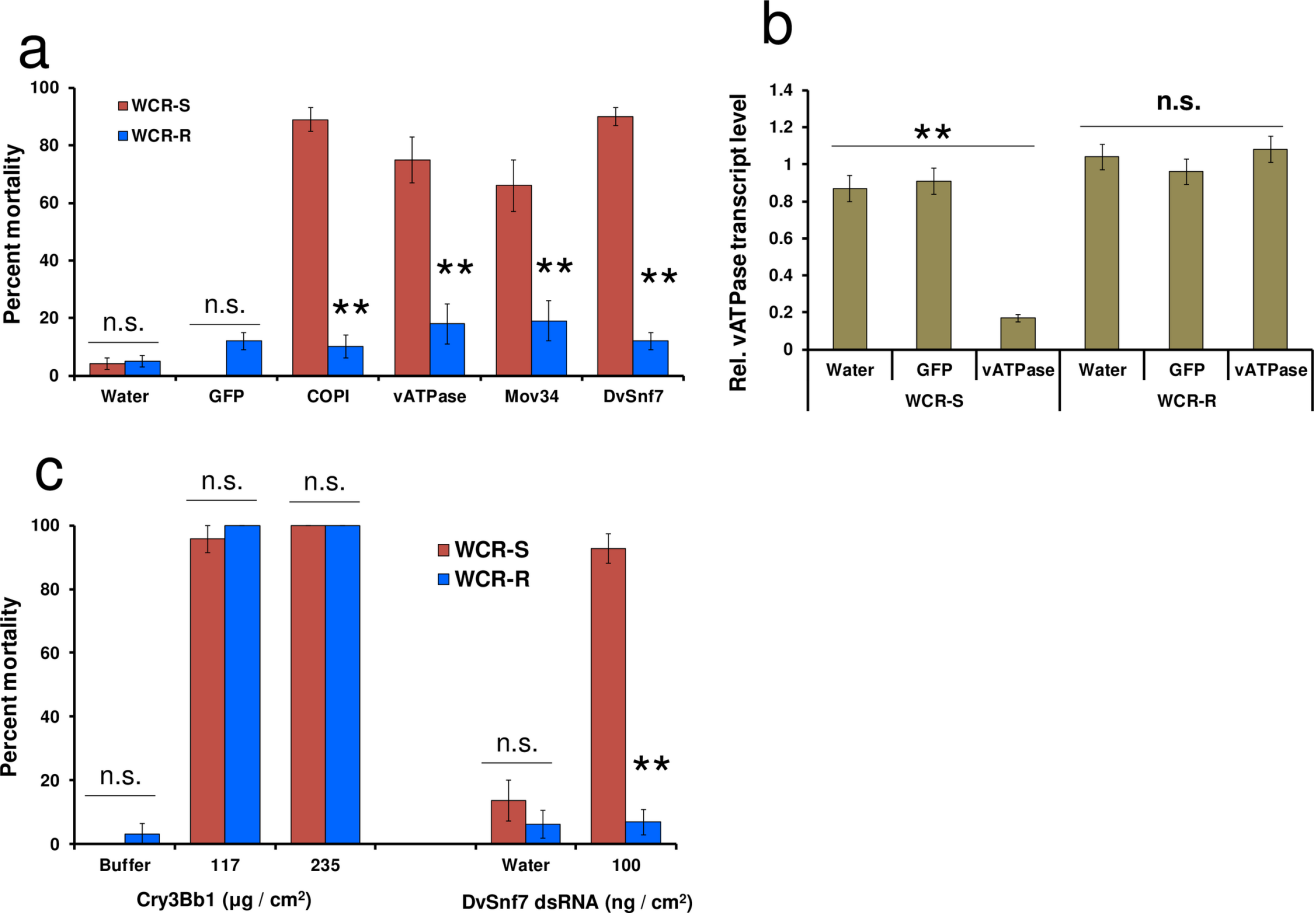
WCR-R is cross resistant to multiple insecticidal dsRNAs but not to Bt Cry3Bb1. (**a**) Percent larval mortality of WCR-R and WCR-S fed artificial diet surface-treated with 300 ng /cm^2^ dsRNA (GFP, COPI β, vATPase A, mov34 or DvSnf7) or water. Each treatment was conducted one to three times, with four replications per trial. WCR-R: *n* = 28–94; WCR-S: *n* = 29–93. (**b**) Relative vATPase A transcript levels in WCR-R and WCR-S larvae fed for 72 hr on artificial diet surface-treated with 300 ng / cm^2^ of dsRNA (vATPase A or GFP) or water. Each treatment consisted of five replications, each consisting of three larvae assayed together with two technical replications. *n* = 15. (**c**) Percent mortality of WCR-R and WCR-S larvae fed artificial diet surface-treated with Cry3Bb1, buffer, DvSnf7 or water. Mortality for Cry3Bb1 was evaluated on day 6 and on day 12 for DvSnf7 dsRNA. Each treatment was conducted once with four replications (*n* = 22–32). All figures show means ± SEM. **p < 0.0001.

To determine if WCR-R larvae were cross resistant to other insecticidal dsRNAs, WCR-R and WCR-S larvae were evaluated against three additional dsRNAs targeting WCR genes in diet overlay bioassays. WCR-R and WCR-S larvae were exposed to artificial diet surface-treated with vATPase A (ATPase subunit A), COPI β (Coatomer Subunit beta), Mov34 (26S proteasome), DvSnf7 or GFP dsRNAs, or water. Mortality of WCR-S larvae exposed to COPI β (89%) (*p* < 0.0001), Mov34 (66%) (*p* = 0.0007), vATPase A (75%) (*p* < 0.0001) or DvSnf7 (90%) (*p* < 0.0001) dsRNA was significantly greater than for WCR-R larvae after 12 d; percent mortality of WCR-R larvae in all treatments was below 20% (Fig 3A). Percent mortality of WCR-R and WCR-S larvae exposed to GFP dsRNA (*p* = 0.967) or water (*p* = 0.746) was not significantly different and remained below 20%. To further assess cross resistance in WCR-R, we measured transcript levels of vATPase A (Fig 3B) and DvSnf7 (S1 Fig) in WCR-R and WCR-S larvae fed diet containing DvSnf7, vATPase A or GFP dsRNA, or water. A significant reduction (5-fold; *p* < 0.0001) in vATPase A transcript levels in WCR-S larvae exposed to vATPase A dsRNA was observed compared to those exposed to water or GFP dsRNA. However, no significant suppression (GFP vs vATPase A; *p* = 0.215 and vATPase A vs Water; *p* = 0.656)) in vATPase A transcript levels was observed in any treatment with WCR-R. As expected, there was a significant reduction (3.6-fold; *p* < 0.0001) in DvSnf7 transcript levels in WCR-S larvae exposed to DvSnf7 dsRNA when compared to larvae exposed to water or GFP dsRNA (S1 Fig). No significant difference (GFP vs DvSnf7; *p* = 0.327 and DvSnf7 vs Water; *p* = 0.127) was found in DvSnf7 transcript levels among WCR-R larvae fed water, GFP or DvSnf7 dsRNA further demonstrating that WCR-R was resistant to DvSnf7 dsRNA.

To determine if WCR-R were also cross resistant to a CRW-active Bt Cry protein, we tested WCR-R and WCR-S larvae against Cry3Bb1 in diet overlay bioassays (Fig 3C). We observed no significant difference (*p* = 1.0) and almost 100% mortality in both WCR-R and WCR-S larvae at both Cry3Bb1 concentrations tested, indicating no cross-resistance between dsRNA and a Bt Cry protein.

### DsRNA uptake, and not degradation, is altered in WCR-R

**Fig 4:**
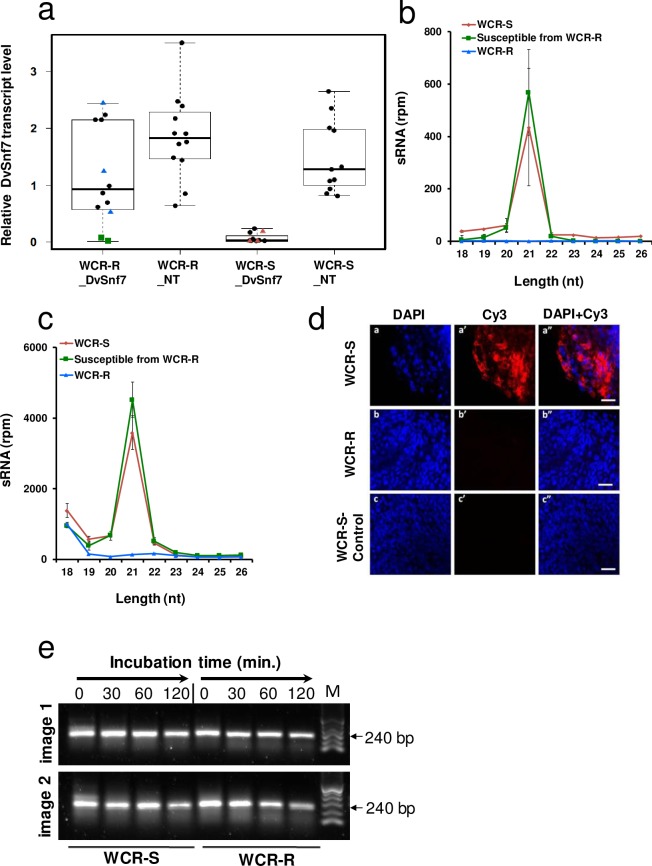
DsRNA uptake, and not degradation, is altered in WCR-R. (**a**) Transcript levels of *DvSnf7* in the carcass of larvae fed either DvSnf7 or non-transgenic (NT) maize roots. Each dot represents the transcript level from a single larva. Larvae represented by blue triangles (3 larvae) and green squares (2 larvae) from WCR-R, and red triangles (3 larvae) from WCR-S were selected for sRNA sequencing (**b**) sRNA reads mapped to the DvSnf7 240-bp dsRNA that were identified from the carcass of WCR-S and WCR-R individual larvae fed DvSnf7 maize roots. The Y-axis is sRNA reads per million (rpm) and the X-axis is the length of sRNA in nucleotides (nt). (**c**) sRNA reads mapped to maize endogenous dsRNA that were identified from the carcass of the individual larvae reared on DvSnf7 maize roots. The same sRNA dataset as for Fig 4B was used for this analysis. (**d**) Uptake of Cy3-labeled DvSnf7 240-mer dsRNA in WCR-S midgut cells (a’), while uptake of Cy3-labeled 240bp DvSnf7 dsRNA was not observed in WCR-R midgut cells (b’). Controls with Cy-3 dye alone did not show intracellular incorporation of Cy3-labeled DvSnf7 240-mer dsRNA (c’); DAPI staining of nuclei was used to visualize midgut cells (a,b,c) and overlaid with Cy3 staining (a”,b”,c”). Representative images of 8–9 midguts per treatment per experiment are shown. Scale Bar: 50 μm. (**e**) Agarose gel image showing intact dsRNA when incubated with midgut juice extract from 10 WCR-R and WCR-S larvae. DvSnf7 dsRNA (1 μg) was incubated with 5 (image 1) or 10 μg (image 2) of total midgut protein extracted from isolated midgut tissue. Aliquots of the incubations were drawn at the times indicated, quenched by ethanol precipitation, and resolved by agarose gel electrophoresis. M = 100 bp DNA Ladder. (b and c) Means ± SEM.

To elucidate the mechanism of WCR resistance to dsRNA-mediated RNAi, we used small RNA (sRNA) sequencing to monitor accumulation of exogenous siRNAs in the gut and carcass of WCR-R and WCR-S larvae fed DvSnf7 maize roots. To select larvae for sRNA sequencing, we measured DvSnf7 transcript levels in both carcass and gut tissues using RT-qPCR. WCR-S larvae that were fed DvSnf7 maize roots had reduced DvSnf7 transcript levels in the carcass as compared to larvae fed NT maize roots (Fig 4A). However, among WCR-R larvae fed DvSnf7 maize roots, two (Fig 4A, green squares) out of 12 larvae exhibited reduced DvSnf7 transcript levels in the carcass compared to larvae fed NT maize roots. This suggests that these two larvae were susceptible to DvSnf7 dsRNA even though they belong to the WCR-R population. Similar results were observed for DvSnf7 expression in gut tissues (S2b Fig). For sRNA sequencing, larvae were divided into three groups 1) WCR-S larvae fed DvSnf7 maize roots with reduced DvSnf7 transcript levels (Fig 4A- red triangles); 2) WCR-R larvae fed DvSnf7 maize roots with DvSnf7 transcript levels (blue triangles) similar to larvae on NT maize; and 3) WCR-R larvae fed DvSnf7 maize roots that had reduced DvSnf7 transcript levels (green squares). Total WCR sRNA reads mapping to the 240 bp DvSnf7 dsRNA sequence expressed in maize roots revealed that DvSnf7-derived siRNAs accumulated in the carcass and gut tissues of WCR-S (Group 1) and DvSnf7 dsRNA-susceptible larvae from WCR-R (Group 3) but not in DvSnf7 dsRNA-resistant larvae from WCR-R (Group 2) (Fig 4B and S2C Fig**).** Total sRNAs sequenced in WCR larvae were also mapped to the maize genome and transcriptome to identify siRNAs derived from numerous long endogenous maize dsRNA transcripts taken up by WCR gut cells[24]. Similar to the DvSnf7 maize siRNA sequencing results, maize long dsRNA-derived siRNAs were only detected in the carcass and gut tissues of WCR-S and DvSnf7 dsRNA-susceptible larvae from WCR-R (Group 3) but not in DvSnf7 dsRNA-resistant WCR-R larvae (Fig 4C and S2D Fig). These data provide further support that WCR-R resistance to dsRNA is not DvSnf7 sequence—or size-specific, but rather a more general dsRNA resistance mechanism. We also looked at the abundance of endogenous 21 bp siRNAs specific from WCR. Interestingly, the expression pattern was similar (*p* = 0.709) in WCR-R and WCR-S larvae (S3 Fig) indicating that biogenesis of endogenous WCR 21 bp siRNA was not affected, and the major components of intracellular RNAi machinery were functional in WCR-R. Altogether, these results suggest that exogenous dsRNA uptake or transport in the gut are specifically affected.

To determine if dsRNA uptake was impaired in WCR-R and WCR-S larval midgut cells, the 240 bp DvSnf7 dsRNA was labeled with Cy3 dye to allow for microscopic visualization. The labeled molecules were then incubated with WCR midgut tissue culture. The 240 bp Cy3-labeled-DvSnf7 dsRNA was localized inside WCR-S midgut cells (Fig 4D; panels a,a,’a”), while no localization of Cy3-labeled 240bp DvSnf7 dsRNA was observed in WCR-R midgut cells (Fig 4D; panels b,b’,b”) indicating that that long dsRNA uptake by midgut cells was impaired in WCR-R.

To assess if enhanced dsRNA degradation is involved with DvSnf7 dsRNA resistance in WCR-R, midgut juice from third instar WCR-R and WCR-S larvae were incubated with DvSnf7 dsRNA for up to 120 min. at 23° C. Our results show no evidence of enhanced DvSnf7 dsRNA degradation in the gut of WCR-R compared WCR-S larvae at any timepoint (Fig 4E).

### The DvSnf7 dsRNA-resistant locus is located on LG 4

**Fig 5:**
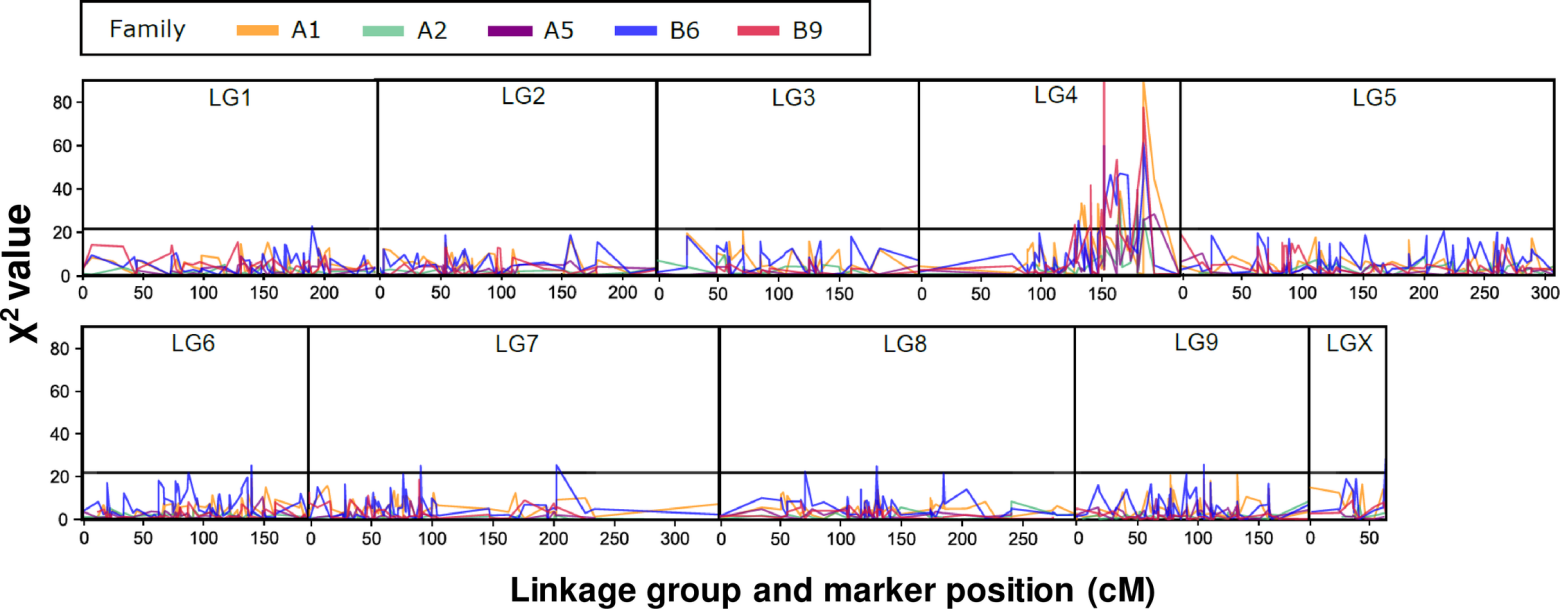
DvSnf7 dsRNA resistant locus is located on LG 4. Reciprocal single parent crosses were made between WCR-R and WCR-S beetles. Families A1, A2, and A5 contained a male WCR-R parent whereas families B6 and B9 contained a female WCR-R parent. F_2_ individuals surviving on DvSnf7 or NT maize from each of five families were genotyped using the WCR SNP genotyping platform. Each panel represents one of the ten WCR linkage groups (LG). Within each LG, markers are ordered based on their genetic map position. The y-axis gives the χ^2^ test statistic between the genotypic counts among F_2_ populations surviving maize expressing DvSnf7 dsRNA or a non-transgenic control. Results from each of the five mapping families are plotted using a separate color following the legend above the figure. A black horizontal line on each panel indicates the Bonferroni threshold of 21.8.

To determine the location of potential DvSnf7 dsRNA resistance gene(s), reciprocal single parent crosses were made between WCR-R and WCR-S beetles (S4 Fig). F_2_ individuals surviving on DvSnf7 maize or NT maize roots from each of five families (A1, A2, A5, B6, and B9) were genotyped using the WCR SNP genotyping platform[25]. χ^2^ tests were performed on genotype counts for the DvSnf7 and NT maize survivors and plotted along the genetic map (Fig 5). We observed a single resistance locus on the right end of linkage group 4 (LG4) in all five mapping families. As observed in the phenotypic assay, the LG4 resistance locus is autosomal, and accordingly, is detected in crosses where either the maternal or paternal parent harbored the resistance allele.

This genotyping data set was also used to interpret inheritance of resistance. The F_2_-informative marker (i.e., parents with contrasting *AA* x *aa* genotypes) with the highest χ^2^ value in each family was used for this analysis. When reared on DvSnf7 maize, the relative fitness of the SS genotype compared to the RR genotype was nearly zero in each family, however the RS heterozygote phenotype did show low fitness levels. Among the five mapping families, RS-relative fitness ranged between 0.019 and 0.157 with an average of 0.076 (S1 Table), suggesting that, as observed in the phenotypic assay, the genetic signature of the inheritance of DvSnf7 dsRNA resistance was at least highly recessive.

## Discussion

Here we describe the first insect colony with field-derived resistance to dsRNA[26]. This DvSnf7 dsRNA-resistant WCR colony can serve as a valuable tool to understand dsRNA resistance in insects and enable the development of effective IRM strategies for dsRNA products. WCR-R larvae had ≥130-fold resistance to DvSnf7 dsRNA, and resistance was autosomal and conferred by a single locus located on the right end of LG4. Additionally, resistance was recessive (typical for most Bt resistance reports) that should decrease the rate of resistance development by killing a higher percentage of heterozygotes thereby increasing refuge effectiveness strategies[26]. We demonstrated previously that Cry3Bb1-resistant WCR are susceptible to DvSnf7 dsRNA[22] and our current results demonstrate that DvSnf7 dsRNA-resistant WCR are susceptible to Cry3Bb1 further confirming the lack of cross resistance between Cry3Bb1 and DvSnf7 dsRNA. Because there is no cross resistance between Cry3Bb1 and DvSnf7 dsRNA, implying different resistance mechanisms that typically segregate independently, it is not surprising that the Cry3Bb1 resistance gene(s) is located on a different chromosome (LG8)[25] compared to the DvSnf7 dsRNA-resistant gene(s) (LG4). Therefore, combining both insecticidal compounds in a single product should be an effective way to manage the risk of resistance, as also has been demonstrated for *Helicoverpa armigera* resistant to Bt Cry1Ac in cotton[27].

Previously we observed that the accumulation of numerous siRNAs derived from ingested long dsRNA in WCR larval tissues, including carcass tissue[24], correlated with suppression of the target gene in those tissues. In this study, sRNA sequencing analysis revealed a lack of siRNA accumulation from both transgenically-expressed DvSnf7 dsRNA and siRNAs from naturally occurring plant long dsRNAs in either the gut or carcass tissues of WCR-R. This demonstrates that the WCR-R gut is unable to uptake labeled dsRNA in contrast to the WCR-S gut, and suggests that long dsRNA uptake by gut cells also is impaired in WCR-R. We cannot rule out the possibility that the mechanism of exogenous dsRNA processing into 21 bp siRNAs is impaired in WCR-R, but this scenario is unlikely because we observed no significant changes in endogenous 21 bp siRNAs between WCR-S and WCR-R. Together these observations indicate that the basic siRNA biogenesis machinery is not affected in dsRNA-resistant insects, but rather that dsRNA uptake is impaired in insect gut cells and is the primary cause of resistance to ingested dsRNA. A reduction in luminal uptake of dsRNA was also shown to be responsible for reduced susceptibility (refractoriness) to dsRNA when the dipteran, *Bactrocera dorsalis*, was challenged with high concentrations of dsRNA within one generation in the laboratory[28], but to our knowledge our study is the first report describing dsRNA resistant insects. These results are not surprising as reduced uptake has been mentioned as a plausible mechanism of resistance to dsRNA[29, 30]. Lack of uptake of dsRNA is a major barrier for targeted RNAi activity and there exists a range of responsiveness to environmental dsRNA in non-target insects in nature due to natural uptake barriers[29].

WCR-R beetles were not sensitive to DvSnf7 dsRNA injected into the hemolymph, suggesting that, in addition to impaired luminal uptake in gut cells, WCR-R may have impaired dsRNA uptake in other cell types and/or impaired systemic spread of RNAi silencing signal that could be long dsRNAs or short sRNAs. The injection-induced RNAi response was also significantly diminished in *Tribolium castaneum* larvae in which clathrin-dependent endocytosis in the midgut was blocked by inhibitors[31]. Furthermore, endocytosis was also shown to be involved in reduced dsRNA midgut uptake in *B*. *dorsalis*[28] and *Leptinotarsa decemlineata*[32]. However, further work is needed to determine if endocytosis is affected in WCR-R and if it has any role in RNAi resistance.

We also observed that WCR-R was cross-resistant to other insecticidal dsRNAs, demonstrating that dsRNA resistance in WCR-R is not sequence-specific. These results are consistent with previous statements postulating the possibility of reduced dsRNA utility in controlling insect pests due to potential cross-resistance between different dsRNA sequences[30, 33, 34]. Additionally, RNAi technology has been considered as a potential tool to manage adult insects including WCR[10–12, 35]. We observed that WCR-R beetles (selected for resistance as larvae) were also resistant to DvSnf7 dsRNA, suggesting that dsRNA targets may not be a viable option for controlling other life stages that were selected for dsRNA resistance as larvae.

Our results emphasize the need to continue to leverage appropriate IRM strategies, including refuge plantings and pyramiding of dsRNA with other active compounds with different modes of action to delay field-evolved dsRNA resistance in insects. Additionally, information provided in this study should also help in optimizing the utility and prolonging the durability of RNAi technologies to control other insect pests.

## Materials and methods

### Establishing a DvSnf7 resistant WCR colony

To promote relatively high WCR beetle densities for DvSnf7 dsRNA selection, a WCR trap crop (comprised of maize and pumpkin) was grown in 2011 near Waterman, IL; approximately 15–20 WCR beetles per corn plant were observed on this trap crop. On May 4, 2012, 3,500 maize kernels expressing DvSnf7 dsRNA (ZM_S295399) were planted in this same field with a 0.4 m row spacing and 15.2 cm between planted kernels in a 21.3 m × 9.1 m area. In addition, non-transgenic (NT) maize was planted in the area surrounding the DvSnf7 maize plants. When plants reached the V4 stage, all putative DvSnf7 plants were assayed for presence of DvSnf7 using Seed Quality Technology (SQT) analysis. For SQT analysis, samples from each plant were collected and processed per MQC (Molecular Quality Characterization) procedures at Monsanto Company (St. Louis, MO)[22]. Plants failing confirmation were removed from the tented plot resulting in approximately 3,360 DvSnf7 dsRNA-expressing maize plants remaining. On June 7–8, 2012, a 24.4 m × 12.2 m WCR beetle escape-proof tent was placed over the DvSnf7 maize plants. WCR beetles collected from this tented plot were used to establish the DvSnf7 dsRNA-resistant colony. WCR beetles collected from NT maize plants were used to establish a parallel susceptible control colony. Both colonies were reared on appropriate maize seedling root mats grown in plastic containers (49.5 x 46.9 x 24.1 cm) in growth chambers at 25° C, 60% RH and L:D 16:8.

### DvSnf7 dsRNA-resistant WCR (WCR-R)

A total of 350 WCR beetles were collected from the tented field plot. Beetles were brought to the Monsanto facility (Waterman, IL) for further rearing and selection. To increase the number of generations per year and thereby accelerate resistance selection in a univoltine insect such as WCR, WCR males collected from the tent were mated with virgin females from the Waterman non-diapausing lab colony (WMND)[22] resulting in a non-diapausing (ND) population (WCR-R). Offspring from this cross were fed NT maize roots and beetles were sib-mated to produce F2 eggs. Neonates from F2, F3, and F4 generations were selected on DvSnf7 maize. In F4, only 91 beetles survived, therefore, generations F5 and F6 were relaxed (fed NT maize) to increase the population[36]. Generations F7-F11 were selected on DvSnf7 maize and thereafter, WCR-R was selected on DvSnf7 maize every other generation.

### Susceptible WCR (WCR-S)

A total of 500 WCR beetles were collected from NT maize plants surrounding the tented field plot and shipped to Monsanto (Waterman, IL). Similarly as above, males collected from the NT maize were allowed to mate with virgin females from the WMND colony to produce a ND population. F1 neonates were fed NT maize and beetles allowed to sib-mate to produce F2 eggs. To validate that the DvSnf7 dsRNA field selection resulted in an enriched DvSnf7 dsRNA-resistant allele frequency, a portion of these F2 neonates were continuously fed DvSnf7 maize. As expected this population collapsed due to high mortality, indicating that the DvSnf7 dsRNA selection in the field led to an increased DvSnf7 dsRNA resistant allele frequency. Individuals from the remaining F2 eggs (WCR-S) were only reared on NT maize and used as a control colony for resistant characterization work.

### Expression of DvSnf7 dsRNA in the maize roots

DvSnf7 dsRNA expression was determined at V1, V2, and V3 developmental stages of DvSnf7 maize (ZM_S295399) when grown in root mats (Fig 1B). Roots from NT maize were used as a control. Samples from roots were collected and submitted for expression analysis using Quantigene[37]. Root samples were collected from 10 different plants at each developmental stage for DvSnf7 and NT maize.

### Test material

All test and control substances, except nuclease free water used for diet and injection bioassays, were produced by Monsanto Co. (St. Louis, MO). Information regarding the dsRNAs used in this study are presented in S2 Table. Annotation for dsRNA sequences are based on the best hit in NCBI BLASTx. Methods for dsRNA synthesis are proprietary. Nuclease free water (UltraPure Invitrogen, Carlsbad, CA) was used in assays with dsRNAs. Cry3Bb1 storage buffer (10 mM sodium carbonate/bicarbonate, 0.1mM EDTA, pH 10.0) was used for assays with Cry3Bb1.

### WCR larvae diet overlay assay

Larval diet-overlay bioassays (including egg storage, incubation, and egg sterilization procedures) used for evaluating inheritance of resistance and cross resistance were conducted as described previously[22]. Briefly, 20 μl of dsRNA, Cry3Bb1, buffer or water was overlaid onto the diet surface of each well of a 96-well plate. For the inheritance of resistance study, neonates were exposed to increasing concentrations of DvSnf7 dsRNA. All bioassays were conducted with neonates (<24 h old), and scored on day 12 for dsRNAs and day 6 for Cry3Bb1. Larvae were considered dead if they were immobile when touched with a fine tipped paintbrush.

### WCR beetle diet overlay and injection assays

Diet used for beetle bioassays contained 12.5 g dry rootworm diet (BioServ # F9760B), 25 ml of de-ionized water, 0.3 g agar, and 62 μl mold inhibitor. The diet mixture was poured into petri plates about 1 mm thick to minimize feeding from all sides. A corer (13 mm diameter) was used to make discs from solidified diet. Two diet discs were placed into each well of a 32-well tray (Frontier Agricultural Science, # RT32W). Eighteen microliters of the appropriate dsRNA concentration or water only were overlaid over each diet disc and allowed to dry in a laminar flow hood for 10–15 min. Ten newly emerged beetles (24–48 h old) were placed in each well and covered with a lid. Diet was made fresh weekly and stored between diet changes at 4˚C. Beetles were moved to a clean tray with fresh food with appropriate substance overlay on Monday, Wednesday and Friday weekly. Trays were kept at 25˚C and 60% relative humidity. After recording mortality data, dead beetles were removed. Mortality was recorded for 16 d.

For injections, beetles (24–48 h old) were exposed to ether for 1 min. WCR-R and WCR-S beetles were injected with one μl (containing 25 ng of dsRNA) of the appropriate solution into the hemolymph behind the rear leg and placed in one well of a 32-well tray containing untreated artificial diet (same as described above) and covered with a lid. Injections were conducted using an aspirator tube assembly with a glass needle attached to it (#A5177-5EA; Sigma-Aldrich). Beetles were moved to new trays with fresh food on Monday, Wednesday and Friday weekly. Mortality was scored on day 10. Trays were kept at 25˚C and 60% relative humidity.

### Collection of material for sRNA sequencing

WCR-R and WCR-S neonates were reared on NT maize roots until late second instar (Fig 2A). Late second instar larvae from WCR-R and WCR-S were divided into two groups: one group was infested on maize seedlings expressing DvSnf7 dsRNA and the other group was infested on NT maize seedlings. Larvae were allowed to feed for three days at which time each larva was dissected to separate gut from the carcass. DvSnf7 transcript levels were determined from carcass tissues (10–12 larvae) and from gut tissues (six larvae) using RT-qPCR (Fig 4A and S2B Fig).

### Quantitative real time PCR

Total RNA was extracted from whole larvae, beetles or dissected larval tissues using TRIzol (Thermo Fisher Scientific) following manufacturer’s instructions. For RT-qPCR analysis, total RNA was treated with TURBO DNA-free DNase (Ambion) and used as a template for real-time RT-PCR using iTaq Universal SYBR Green One-Step Kit (Bio-Rad) as described previously[17]. Primers from two housekeeping genes, *actin* and/or *tubulin* (endogenous controls) were used for normalization. Primers used to evaluate the transcript levels of all genes are provided in S3 Table.

### sRNA sequencing

Small RNA (sRNA) library preparation and sequencing were conducted as described previously[38] using Illumina TruSeq Small RNA Sample Preparation kits and the Agilent Bioanalyzer High Sensitivity DNA kit following manufacturer’s instructions. Size-selected pools were quantitated using RT-qPCR and sequenced on two lanes of an Illumina HiSeq 2000 NGS system per the manufacturer’s instructions to generate 50 bp single-read sequences. sRNA parsing and analysis was conducted as described previously[24]. To investigate DvSnf7 dsRNA processing into sRNAs after larval feeding, parsed sRNAs were mapped to the DvSnf7 240 bp dsRNA sequence. sRNAs were also mapped to the maize genome (in-house B73, assembly v2) since endogenous maize long dsRNAs can be taken up by WCR larva while feeding on maize roots and processed within the insect to sRNAs[39].

### Midgut tissue culture assay for uptake of labeled dsRNA

Cy3-labeling of the DvSnf7 240 bp dsRNA, and confirmation of labeling, was conducted as described previously[17]. Midguts dissected from second-instar WCR-R and WCR-S larvae under aseptic conditions were used for tissue culture assays to determine uptake of labeled dsRNA as described previously[17]. Images were captured using a 550 nm (Cy3) and 360 nm (DAPI) laser for excitation and 570 nm (Cy3) and 450–460 nm (DAPI) for emission filter sets, under a confocal microscope. Scanned images were processed using LSM (Carl Zeiss AIM; version 4.2) and Adobe Photoshop (CS5 software; version 12.0 x32) software.

### dsRNA midgut stability assay

Extraction of total midgut proteins was conducted as described previously[40]. Midguts were excised from 10 third instar WCR-S and WCR-R larvae and homogenized in cold 150 mM NaCl. Solid material was separated from soluble material by centrifugation at 14,000 g for 10 min. at 4˚C twice. Protein concentration was determined using the Bradford method[41]. DvSnf7 dsRNA (1 μg) was incubated at 23˚C with five or 10 μg of total protein extracted from isolated midgut tissue. Aliquots of the reactions were drawn at 30, 60, and 120 min. and quenched by ethanol precipitation. Recovered dsRNAs were resolved by agarose gel electrophoresis.

### Collection of materials for resistance mapping

Ten single-parent crosses were initiated between WCR-R and WCR-S beetles (S4 Fig). To address the potential for sex biased inheritance of resistance, approximately 50% of the crosses included a male parent from WCR-R and a female parent from WCR-S, while the other half were the reciprocal. Each adult pair was reared in a small cage (Pioneer plastics, catalog # 036C) and provided artificial diet (BioServ # F9760B) and a petri dish containing soil for oviposition. From each cross, F_1_ eggs were collected, reared to adulthood on NT maize roots, and allowed to randomly sib-mate. F_2_ neonates from each family where randomly assigned to a treatment or control group and those from the treatment group were reared on DvSnf7 maize roots, whereas the control larvae were fed NT maize roots. Five F_2_ populations with sufficient insect numbers were selected for phenotypic screening and genotyping. These included three families where the male parent was from WCR-R (A1, A2, and A5), and two where the female parent was from WCR-R (B6 and B9). All larvae surviving to adulthood were collected and saved at -80° C for genotyping.

### Genotyping, QTL mapping, and genetic map repair

All genotyping methods, including DNA extraction, target amplification, and genotyping by sequencing were conducted as previously described[25]. No modifications were made to these processes except that amplicon sequencing was performed on an Ion Proton DNA sequencer (ThermoFisher Scientific) instead of an Ion PGM. The WCR platform contains 1150 biallelic SNP markers, among which 770 have known map positions on one of the 10 WCR linkage groups [25]. To identify markers associated with DvSnf7 dsRNA resistance we performed χ^2^tests of independence between genotypic counts among treatment and control F_2_ survivors. Across all five families 2657 χ^2^ tests were performed, each with 2 degrees of freedom, resulting in a Bonferroni significance threshold of χ^2^ ≥ 21.8. Initial inspection of the χ^2^results indicated that DvSnf7 resistance was strongly associated with linkage group 4 (LG4). However, many of the markers on LG4 most strongly associated with the trait were separated by > 50 cM, while the intervening region included markers that did not respond to DvSn7 dsRNA selection. At face-value, this would suggest two unlinked DvSnf7 dsRNA-resistance loci on LG4. This led us to reassess the construction of LG4 reported previously[25]. The LG4 map was a consensus of six component maps. Five of the six component maps had an inverted order in the middle of LG4 when compared to the consensus, while the sixth matched the LG4 marker order reported previously[25]. We removed this last map and reran MergeMap[42] to produce a new consensus (S4 Table). This new consensus map retains the inverted order that matches five of the original six component maps.

To test if this new LG4 map was an improvement over the older map we compared their relative likelihoods on three segregating F_2_ populations. We first computed the likelihood of the marker orders given the marker data from the three mapping populations used previously[25]. To accomplish this we calculated the log-likelihood (*LL*) of the recombination fraction (equation 2.17 ^41^) specified by the genetic distances in either map pairwise for all LG4 markers for both the old and new maps. Within each mapping population, the old and new map *LL*s were summed to give a joint log-likelihood (*JLL*). Finally, the relative likelihood (*RL*) of the new map vs. the old map was calculated as follows, $RL=e^{\left({JLL}_{new\;map}-{JLL}_{old\;map}\right)}$. Under this framework, the relative likelihoods of the new map were found to be 7.4x10^77^, 5.6x10^124^, and 4.2x10^18^ greater than the old map for the three mapping families, respectively. This gives strong support for the new LG4 map order being a better fit for the segregation data in these populations than the old one reported here[25]. For this reason, we used the new LG4 map order throughout this manuscript.

### Inheritance of resistance

To estimate the dominance of the resistance locus we selected the most significant F_2_-informative marker (i.e. parental genotypes *AA* x *BB*) on LG 4 for each population. Among the F_2_ control populations, each of these markers was consistent with the expected 1:2:1 *RR*:*RS*:*SS* Mendelian segregation ratio (all χ^2^p-values > 0.05). However, among the survivors from the DvSnf7 dsRNA-treated populations these ratios were heavily skewed toward the *RR* genotype. By setting the relative fitness of the *RR* genotype to 1, we can estimate the relative fitness of the *RS* genotype (*ωRS*) as *ωRS* = *RS/(*2*RR)*, where *RS* and *RR* are the counts of *RS* and *RR* genotype among survivors of the DvSnf7 dsRNA treatment. We multiplied *RR* by 2 because we can infer from the 1:2:1 ratios in the control populations that *RS* counts should have been approx. double the *RR* count prior to DvSnf7 dsRNA selection. S5 Table gives the treatment population genotype counts and relative fitness of the *RS* genotype (*ωRS*) for each mapping family along with 95% confidence limits for *ωRS* based on 10^5^ bootstrap resamples of the data. Because the relative fitness of the *SS* genotype is essentially zero, we can interpret *ωRS* as an estimate of the dominance of the *R* allele with respect to the *S* allele, which on average is 0.076.

We also made reciprocal crosses with WCR-R and WCR-S beetles to evaluate dominance of resistance. F1 progeny from reciprocal crosses between WCR-R and WCR-S populations (WCR-R ♀ x WCR-S ♂ and WCR-R ♂ x WCR-S ♀) were evaluated in diet bioassay along with WCR-R and WCR-S. Each cross contained 80 males and 80 females. Separate cages housed WCR-R and WCR-S beetles with similar number of males and females collected from the same generation that were used for making reciprocal crosses. Degree of dominance (D) was calculated using, D = (2X2—X_1_—X_3_) **/ (**X_1_ –X_3_), Where X_2_ is the mean of the LC_50_ values of reciprocal crosses and X_1,_ X_3_ are LC_50_ values of resistant and susceptible populations respectively[23]. As LC_50_ values for WCR-R could not be determined, the highest concentration (500 ng dsRNA / cm^2^) used for resistant insects was used in the calculations. Value of D ranges from -1 to 1, where D = 1 indicates complete dominance; 0 < D < 1 indicates incomplete dominance; -1 < D < 0 indicate incomplete recessive and D = -1 indicates complete recessive.

### Statistical analysis

All analyses described below were conducted using SAS[43]. One-way ANOVA was performed on the DvSnf7 dsRNA expression level of 10 plants from each developmental stage. The analysis showed the homogeneity on log scale among all four treatments. No significant difference was detected among three maize developmental stages, and non-transgenic maize showed a negligible level (Fig 1B). The SAS procedure Proc Probit was applied for the estimation of the dose response curve assuming the logistic model for each treatment separately (Fig 2A). A Goodness-Of-Fit test was based on the likelihood ratio test. Results are listed in Table 1 including the estimated slope and LC_50_ values, and fitness of the model. Natural mortality was also considered in the analysis with Option OPTC. One-way ANOVA was performed on expression of DvSnf7 transcript in beetles (ng/cm^2^) and comparisons were made using a *t*-test for each colony (Fig 2C). A generalized linear model was applied to the mortality data, assuming binomial distribution with a logit link function. The model includes the effect of bio-rep and the treatment. Two biological replicates represent two experimental times, and no difference was detected. Therefore, the treatment effects were pooled across two biological replicates (Fig 2D). One-way ANOVA was performed on relative transcript levels of DvSnf7 in beetles assuming a heterogeneous variance for the WCR-S DvSnf7 dsRNA treatment due to low variance. Comparisons were made by using a *t*-test for each colony (Fig 2E). The observed WCR mortality for dsRNA cross resistance assays was analyzed with a logistic regression on six diet treatments for each of WCR-S and WCR-R using SAS GLIMMIX. Pearson’s Chi-Square divided by the degrees of freedom showed a value of 0.99, indicating a good fit of the model, and that the interaction between WCR colony and diet treatment and the variation among three assays and 12 plates in data, if any, should be negligible. The significance of the pairwise comparisons is determined on a logit scale, and the treatment mean mortalities along with their standard errors are the least square means using the inverse link function (Fig 3A). One-way ANOVA was performed on relative vATPase A transcript levels assuming a heterogeneous variance for the WCR-S vATPase A treatment due to low variance. Comparisons were made using a *t*-test for each colony (Fig 3B). Fisher’s exact test was performed for the comparisons between two colonies for each treatment due to mortality being close to 100% or 0% (Fig 3C). One-way ANOVA was performed on DvSnf7 transcript levels with the observed heterogenous variation considered in the model. Comparisons were made using a *t*-test between treatments for each colony (S1 Fig).

## Supporting information

S1 Fig Relative DvSnf7 transcript levels in WCR-R and WCR-S larvae exposed to dsRNA (DvSnf7 or GFP) or water. (DOCX)

S2 Fig Assay to determine DvSnf7 transcript levels in WCR-R and WCR-S. (DOCX)

S3 Fig WCR 21 bp-long siRNAs identified in the carcass of WCR-S and WCR-R larvae. (DOCX)

S4 Fig Mating design used to map resistance and number of F_2_ survivors. (DOCX)

S1 Table Relative fitness of the *RS* genotype (*ωRS*) at the F_2_-informative marker most closely linked with resistance in each family. (DOCX)

S2 Table Sequence and size of dsRNAs used in this study. (DOCX)

S3 Table Primer sequences used for qRT-PCR analysis. (DOCX)

S4 Table List of each marker along with its estimated position in centimorgans on linkage group 4. (XLSX)

S5 Table Chi-Squared test results for association with DvSnF7 resistance is given for every marker in every mapping family. (XLSX)

S1 Data (XLSX)
