# Supplementary material for: Development and characterization of the first dsRNA-resistant insect population from western corn rootworm, Diabrotica virgifera virgifera LeConte
Source: PLoS One. 2018 May 14;13(5):e0197059. doi: 10.1371/journal.pone.0197059 (PMC5951553; doi:10.1371/journal.pone.0197059)
Supplement: S1 Fig — (DOCX) [file pone.0197059.s001.docx]

**S1 Fig. Relative DvSnf7 transcript levels in WCR-R and WCR-S larvae exposed to dsRNA (DvSnf7 or GFP) or water.** These insects were the part of the same assay as in Fig. 3B**.** These results show significant reduction in DvSnf7 transcript levels in WCR-S larvae fed artificial diet surface-treated with DvSnf7 dsRNA when compared to larvae fed water or GFP dsRNA. No significant difference was found in WCR-R larvae fed water, GFP or DvSnf7 dsRNA. Standard error for each treatment was calculated from 5 replications, each consisting of three larvae assayed together with two technical replications. *n*=15. Mean ± SEM. ***P* < 0.0001.
