# Supplementary material for: Development and characterization of the first dsRNA-resistant insect population from western corn rootworm, Diabrotica virgifera virgifera LeConte
Source: PLoS One. 2018 May 14;13(5):e0197059. doi: 10.1371/journal.pone.0197059 (PMC5951553; doi:10.1371/journal.pone.0197059)
Supplement: S2 Fig — (DOCX) [file pone.0197059.s002.docx]

**S2 Fig.** (**a**) Assay design to determine DvSnf7 transcript levels in WCR-R and WCR-S. (**b**) Transcript levels of the DvSnf7 gene in the gut of larvae exposed to either DvSnf7 or non-transgenic (NT) maize roots. Each dot or triangle represent transcript levels from an individual larva. Larvae represented by blue and purple triangles from WCR-R and red triangles from WCR-S were selected for sRNA sequencing. Two larvae circled are the same larvae represented by purple triangles in Fig. 4A (**c**) sRNA reads mapped to the DvSnf7 240-mer dsRNA identified from the gut of individual larvae reared on DvSnf7 maize roots. Y-axis is sRNA reads per million (rpm) and x-axis is nucleotide length (nt). (**d**) sRNA reads mapped to maize dsRNA identified from the gut of individual larvae reared on DvSnf7 maize roots. Same sRNA data as of 2C Fig was used for this analysis.
