# Supplementary material for: Development and characterization of the first dsRNA-resistant insect population from western corn rootworm, Diabrotica virgifera virgifera LeConte
Source: PLoS One. 2018 May 14;13(5):e0197059. doi: 10.1371/journal.pone.0197059 (PMC5951553; doi:10.1371/journal.pone.0197059)
Supplement: S3 Fig — (DOCX) [file pone.0197059.s003.docx]

**S3 Fig.** WCR 21 bp-long siRNAs identified in the carcass of WCR-S and WCR-R larvae. Y-axis is sRNA reads per million (rpm). No significant difference was observed across both treatments. Each treatment consisted of 3 larvae sequenced individually. Mean ± SEM. WCR-S vs. WCR-S; *P* = 0.709. The same sRNA sequencing data as of Fig. 4B and 4C was used for this analysis except that siRNAs mapped to sequences from the maize genome and to the DvSnf7 240-mer dsRNA sequence expressed in maize were excluded.
