# Supplementary material for: Development and characterization of the first dsRNA-resistant insect population from western corn rootworm, Diabrotica virgifera virgifera LeConte
Source: PLoS One. 2018 May 14;13(5):e0197059. doi: 10.1371/journal.pone.0197059 (PMC5951553; doi:10.1371/journal.pone.0197059)
Supplement: S4 Fig — (DOCX) [file pone.0197059.s004.docx]

**S4 Fig. Mating design used to map resistance and number of F_2_ survivors**. Three generations of insects were reared to conduct the mating experiment. The F_1_ generation was sib-mated and eggs from the F_2_ generation were randomly separated into two groups. Insect survivors of non-transgenic and DvSnf7 maize are tallied in tables at the bottom of the Figure.
