# Supplementary material for: Development and characterization of the first dsRNA-resistant insect population from western corn rootworm, Diabrotica virgifera virgifera LeConte
Source: PLoS One. 2018 May 14;13(5):e0197059. doi: 10.1371/journal.pone.0197059 (PMC5951553; doi:10.1371/journal.pone.0197059)
Supplement: S1 Table — (DOCX) [file pone.0197059.s005.docx]

**S1 Table:** Relative fitness of the *RS* genotype (*ωRS*) at the F_2_-informative marker most closely linked with resistance in each family. Families A1, A2, and A5 had a male parent whereas families B6 and B9 had a female parent from WCR-R. *RR*, *RS*, and *SS* are the genotypic counts of resistant parent homozygote, heterozygote, and susceptible parent homozygote genotypes within DvSnf7 dsRNA selected F_2_ populations.

| Family | Marker | *RR* | *RS* | *SS* | *ωRS* | lower conf. int. | upper conf. int. |
| --- | --- | --- | --- | --- | --- | --- | --- |
| A1 | CRW2396 | 52 | 2 | 1 | 0.019 | 0 | 0.051 |
| A2 | CRW1774 | 46 | 4 | 0 | 0.043 | 0.010 | 0.095 |
| A5 | CRW2051 | 41 | 2 | 0 | 0.047 | 0 | 0.066 |
| B6 | CRW1893 | 67 | 21 | 1 | 0.157 | 0.087 | 0.246 |
| B9 | CRW1887 | 107 | 24 | 0 | 0.112 | 0.070 | 0.168 |
| Average | NA | NA | NA | NA | 0.076 | 0.052 | 0.096 |
