# Supplementary material for: Development and characterization of the first dsRNA-resistant insect population from western corn rootworm, Diabrotica virgifera virgifera LeConte
Source: PLoS One. 2018 May 14;13(5):e0197059. doi: 10.1371/journal.pone.0197059 (PMC5951553; doi:10.1371/journal.pone.0197059)
Supplement: S2 Table — (DOCX) [file pone.0197059.s006.docx]

**S2 Table:** Sequence and size of dsRNAs used in this study

| **Target ID** | **Size (bp)** | **Sequence** |
| --- | --- | --- |
| DvSnf7 | 240 | GCAAAGAAAAAUGCGUCGAAAAAUAAAAGAGUUGCACUCCAAGCCCUCAAAAAGAAGAAACGAUUGGAAAAGACCCAACUACAAAUAGAUGGAACCCUUACAACUAUUGAAAUGCAGAGGGAAGCCCUCGAAGGAGCUAGCACAAAUACUGCUGUAUUAGAUUCUAUGAAAAAUGCUGCAGAUGCCCUUAAGAAAGCUCAUAAGAAUUUGAAUGUAGAUGAUGUUCACGAUAUCAUGGAU |
| vATPase (Subunit A) | 232 | GAGAAGCCUGGCAAUUUCCAAGGUGAUUUUGUCCGUUUCUGCCAGAGAUGCUUUACCUACCAGCUGCACAAUUUCGGCUAGAUCAUCUUCUUCCUGAAGAAUUUCCUUAACUUUGGUUCUAAGAGGAAUAAACUCUUGGAAGUUUUUGUCAUAAAAGUCGUCCAAUGCUCUUAAAUAUUUGGAAUAUGAUCCAAGCCAGUCUACUGAAGGGAAGUGCUUACGUUGGGCAAGC |
| GFP | 351 | GCCAGAUACCCAGACCACAUGAAGCAGCACGACUUCUUCAAGUCUGCCAUGCCAGAGGGUUACGUGCAGGAGAGAACCAUCUUCUUCAAGGACGACGGUAACUACAAGACCAGAGCCGAGGUGAAGUUCGAGGGUGACACCCUGGUGAACAGAAUCGAGCUGAAGGGUAUCGACUUCAAGGAGGACGGUAACAUCCUGGGUCACAAGCUGGAGUACAACUACAACUCUCACAACGUGUACAUCAUGGCCGACAAGCAGAAGAACGGUAUCAAGGUGAACUUCAAGAUCAGACACAACAUCGAGGACGGUUCUGUGCAGCUGGCCGACCACUACCAGCAGAACACCCCAAUC |
| COPI β  (Coatomer Subunit beta) | 242 | GCAGGCUGAUAGCACUUAAGGAGCUUCCUAAUCACGAAAGAAUUCUGCAGGAUUUAGUUAUGGACAUACUGAGAGUACUCUCUGCUCCUGACUUAGAAGUCCGCAAGAAGACUUUAAGUCUAGCCCUUGAAUUAGUCUCUUCACGGAACAUAGAAGAAAUGGUAUUAGUAUUAACAAAGGAAGUGAGUAAAACGGUAGACAGUGAACAUGAGGAUACAGGAAAGUACAGGCAAUUGUUAGUC |
| Mov34 (26S proteasome) | 240 | UgUagaagaagUacacgAUGacggUUcaccUacaUcUaaaacaUUUgagcAUGUUccUagUgaaaUcggagcUgaggaagcUgaagaagUUggUgUUgaacacUUaUUgagagaUaUUaaagacacaacUgUUggUacUUUgUcacagagaaUaacgaaUcagcUUUUagggUUaaaaggUcUccaUUcacagUUacgUgaUaUUagggacUaUUUggUacaagUgUgUggUaaccagcU |
