# Supplementary material for: Development and characterization of the first dsRNA-resistant insect population from western corn rootworm, Diabrotica virgifera virgifera LeConte
Source: PLoS One. 2018 May 14;13(5):e0197059. doi: 10.1371/journal.pone.0197059 (PMC5951553; doi:10.1371/journal.pone.0197059)
Supplement: S3 Table — (DOCX) [file pone.0197059.s007.docx]

**S3 Table**: Primer sequences used for qRT-PCR analysis.

| **Gene name** | **Primer sequences for qRT-PCR** | **Product length (bp)** |
| --- | --- | --- |
| *Actin* | Forward: GGTGGGTGGAGCATAGTGAC  Reverse: CTGGTCCATTTTGCCAATTC | 155 |
| *Tubulin* | Forward: CCAAGAGAGCTTTCGTCCAC  Reverse: TTCAGCTCCTTCACCCTCAC | 146 |
| *vATPase A* | Forward: ATCTACGTCGGTTGCGGAGAAAGA  Reverse: TTAGATGTGTTGGCGACCAATGCG | 134 |
| *DvSnf7* | Forward: CCGACGATCTGGATGACGA  Reverse: TTACGAGGCCCAGGCTTCC | 206 |
